# Psychiatric Symptoms of Children and Adolescents With Mitochondrial Disorders: A Descriptive Case Series

**DOI:** 10.3389/fpsyt.2021.685532

**Published:** 2021-07-20

**Authors:** Elise Riquin, Thomas Le Nerzé, Natwin Pasquini, Magalie Barth, Clément Prouteau, Estelle Colin, Patrizia Amati Bonneau, Vincent Procaccio, Patrick Van Bogaert, Philippe Duverger, Dominique Bonneau, Arnaud Roy

**Affiliations:** ^1^Department of Child and Adolescent Psychiatry, University Hospital of Angers, Angers, France; ^2^University Angers, [CHU Angers], LPPL EA4638, Angers, France; ^3^University Angers, [CHU Angers], INSERM, CNRS, MITOVASC, SFR ICAT, Angers, France; ^4^Department of Genetics and National Reference Center for Mitochondrial Disorders, University Hospital of Angers, Angers, France; ^5^Department of Pediatric Neurology, University Hospital of Angers, Angers, France; ^6^Reference Center for Learning Disabilities, University Hospital of Nantes, Nantes, France

**Keywords:** mitochondrial disorders, child, adolescent & youth, psychiatric symptom, anxiety, depression

## Abstract

**Background:** Mitochondrial disorders (MD) are a group of clinically heterogeneous genetic disorders resulting from dysfunction of the mitochondrial respiratory chain. Cognitive impairment is a common feature in adults with MD and psychiatric symptoms are associated with MD in up to 70% of the adult population. The aim of this study is to describe the psychiatric profile in children and adolescents with MD by focusing on the description of psychiatric symptoms.

**Methods:** A cohort of 12 children and adolescents was prospectively recruited between February 2019 and February 2020 in the Reference Center for Mitochondrial Disorders of Angers (France). Participants and their parents completed an anamnestic form to provide socio-demographic data and completed the Global Assessment of Functioning scale, the Brief Psychiatric Rating Scale, the Child Depression Inventory, the Revised Children's Manifest Anxiety Scale, and the Conner's Rating Scale to evaluate the inattention/hyperactivity symptoms as well as the Quality of Life scale.

**Results:** Four children (33.3%) were diagnosed with depressive symptoms. With regarding to anxiety, 6 children (50%) reported anxiety issues during the psychiatric interview and 3 children (25%) were suffering from anxiety according to the RCMAS scale. Compared to other children with chronic illnesses, the individuals in our cohort reported a lower overall quality of life score and lower scores in physical and social subscales.

**Conclusion:** Our study shows that MD can lead to psychiatric disorders in children and adolescents, in particular anxiety and depression, as well as poor quality of life. This highlights the need for regular psychiatric assessments in individuals with developing brains, such as children and adolescents. We do not, however, have data regarding the neuropsychological profile of this population.

## Introduction

Mitochondrial disorders (MD) are a group of clinically heterogeneous genetic disorders resulting from dysfunction of the mitochondrial respiratory chain (1). In children (<16 years old), the estimated prevalence of MD ranges from 5 to 15 cases per 100,000 individuals (1). MD can be caused either by mutations in the mitochondrial DNA (mtDNA) or in nuclear genes that encode proteins involved in mitochondrial function (2). MD often involve multiple organ systems and typically affect organs that require the greatest amount of energy (i.e. brain, heart, muscles, kidneys) (3–5). The main central nervous system (CNS) symptoms of MD include epilepsy, hearing loss, visual impairment, intellectual disability, fluctuating encephalopathy, stroke-like episodes, ataxia, and spasticity.

Brain dysfunction in MD can also result in neuropsychological or psychiatric disturbances including mentation, mood, and behavioral disorders, but few studies deal with this aspect of MD (6, 7). Cognitive impairment is also a common feature in adults and children presenting with MD, such as MELAS (Mitochondrial Encephalopathy Lactic Acidosis, and Stroke-like episodes) syndrome (8) and psychiatric symptoms are associated with MD in up to 70% of the adult population (9, 10). Similarly, a recent article (11) suggested that mitochondrial activity could regulate the availability of GABA, an inhibitory neurotransmitter, in neurons thus leading to social deficits that can be mediated by the modulation of GABA levels. The main psychiatric symptoms observed in adults with MD are depression, psychosis, cognitive deterioration, anxiety, and bipolar disorders as well as frontal lobe syndromes.

It is crucial to consider this aspect of MD in the pediatric population because the disease occurs in developing brains and may not only have a significant impact on developmental trajectories, but may also lead to (neuro)-psychological disturbances that can affect the quality of life.

Children and adolescents affected with MD may have psychiatric symptoms and neuropsychological impairment that are similar to those observed in adults, thus highlighting the importance of considering them as key clinical signs for the diagnosis of MD in pediatric practice as well (12). In the pediatric population, psychiatric features seem to associate depression with behavioral disorders as well as psychomotor and developmental delays (13), although assessments of cognitive function show some variation (14). In addition, as the disease progresses, many children experience a decline in cognitive function (15).

As the literature is still slight on this field, the aim of this study was to better describe the psychiatric features in children and adolescents with MD, in accordance with the ICD-10 International Classification of Diseases.

## Patients and Methods

### Study Design

We conducted a single-center, cross-sectional descriptive, and analytical exploratory study consisting of an evaluation of children and adolescents affected with MD, conducted by a psychiatrist, a neuropsychologist, and a pediatrician. The study protocol was approved by the French Committee for the Protection of Subjects involved in Biomedical Research. Once complete information about the study had been provided, all subjects (children and their parents) gave their written consent to participate in the study. All patient data were anonymized.

### Individuals

Twelve children and adolescents were prospectively recruited over a 1 year period (2019–2020). They all received information about the study either orally during a routine consultation with a pediatrician (MB) or by post. The inclusion criteria were that the subject was: (i) a child between 6 and 17 years of age, affected with a MD defined by the presence either of a mutation known to be involved in mitochondrial cytopathy or of an abnormality in the mitochondrial respiratory chain; (ii) registered with a social security scheme; and (iii) had accepted (both child and the parents) to participate in the study and to had signed the consent form.

Exclusion criteria were: (i) the child's refusal to participate in the study; (ii) the refusal of parents that their child participate in the study; (iii) the inability of the child to answer questions to complete the questionnaires (e.g., non-communicative child); and (iv) if the child had been previously recruited in an intervention research.

**Figure 1:**
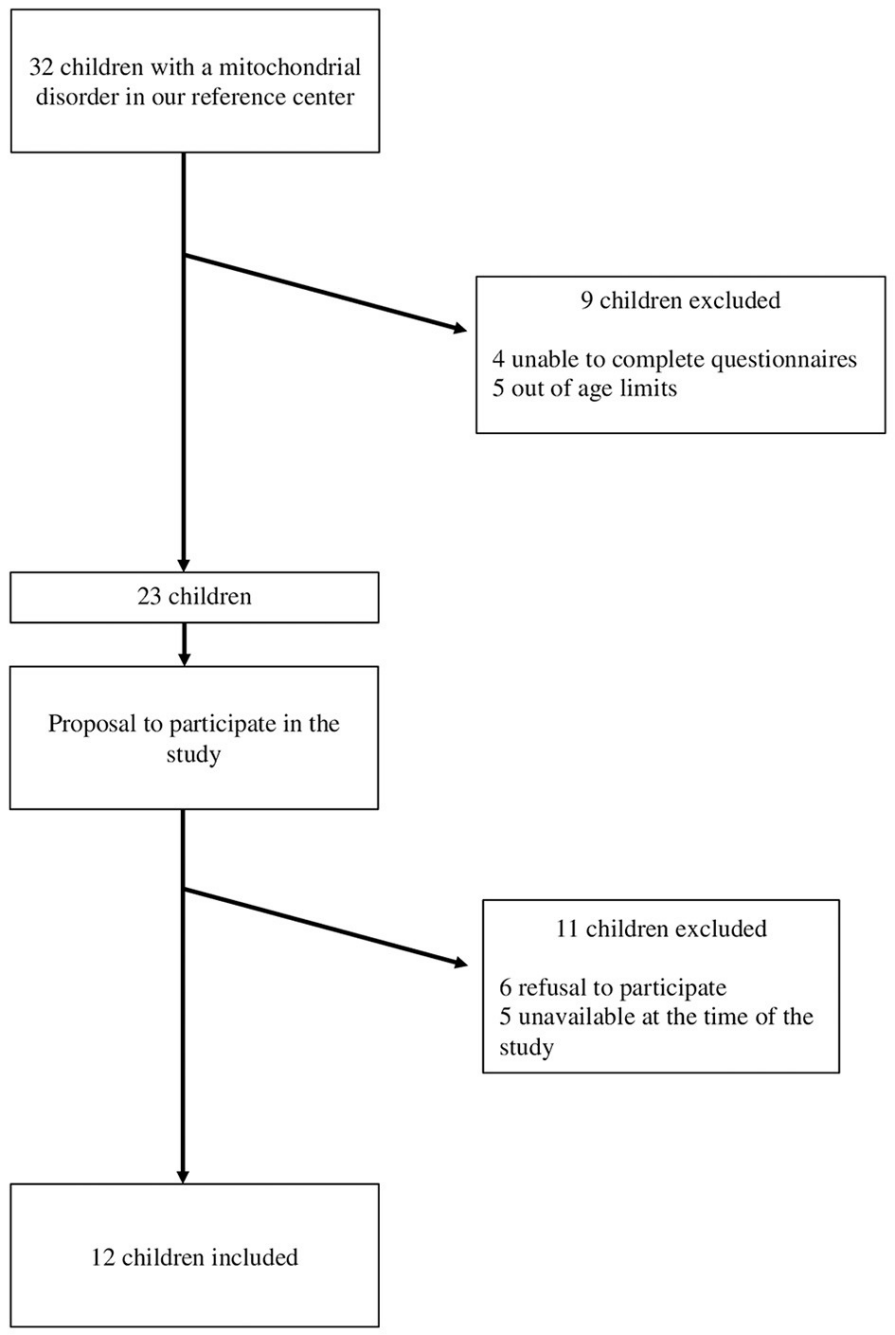
Study flow chart.

The research protocol was presented orally to the potential participants and their families. Those who agreed to participate and who met the inclusion criteria received the consent forms to sign, and they were included in the study by the pediatrician. The flow chart of the study is shown in Figure 1.

The children or their parents filled out a form to determine their socio-demographic and anamnestic data, sex, and age at the time of diagnosis. The diagnosis of mitochondrial pathology (i.e. the genetic variant responsible for the mitochondrial disorder or abnormalities of the mitochondrial respiratory chain) has been specified by the pediatrician. These data were collected in a file by the pediatrician involved in the inclusions.

Data on the medical and psychiatric history and age of onset of psychiatric symptoms were also collected, as well as data on the child's clinical status at the time of consultation. All individuals were diagnosed as having mitochondrial respiratory chain complex defects through biochemical enzymatic assays on muscle tissue samples and met the modified MD criteria proposed by Bernier et al. (16). Lactic acidosis was classified as mild, moderate, or severe, according to the increase from normal reference values (≥2, ≥3, or ≥4-fold increase, respectively, *N* = 0.5–2.2 mmol/L.).

The psychiatric evaluation was based on an interview including the completion of questionnaires with the child and his or her family. The diagnosis of possible psychiatric disorders was made in accordance with the International Classification of Diseases (10th Revision) (ICD-10) (17). The following assessment scales were used:

#### The Children's Global Assessment Scale

This scale is designed to assess the general functioning of a subject along a continuum of three domains (i.e., psychological, social adaptation, and activity) (18). The CGAS is graded along a continuum in increments of 10. The gradation ranges from 1 to 10 for the sickest individuals, to 90–100 for individuals who are virtually symptom-free and functioning satisfactorily in their social environment or family. This scale can be used regardless of the patient's age with a version adapted to individuals aged 3 to 18.

#### The Brief Psychiatric Rating Scale

The BPRS was first published in 1962 by Overall and Gorham (19) and a French version has been available since 1967 (20). The BPRS is the most widely used instrument of this type in the international literature (21). This is an 18-item scale covering every aspect of overt psychopathology. The global nature of the description adopted for each item corresponds to the usual way in which clinicians typically understand symptoms. Each item corresponds to a category of intensity or frequency numbered from 1 (absence) to 7 (maximum intensity or frequency). For most questions, scales 2 to 3 define a range of non-pathological symptoms; while scales 4 to 7 assess pathological deviation. Thus, the scores can vary from 18 to 126; lower scores indicate less severe psychopathology and scores above 72 define pathological deviation.

#### The Children's Depression Inventory

This self-assessment questionnaire rates the severity of depression in school-age children and adolescents from 7 to 17 years old (22). However, the CDI, which has been adapted for children from the Beck's Depression Inventory 21 (BDI-21) (23), is not a diagnostic tool and must be complemented by a clinical assessment. Test-retest fidelity was 0.43 after 1-month interval. Cronbach alpha was 0.70. About the construct validity, Structural factor analysis reveals a unidimensional scale with the first factor accounting for 63.7% of the variance. Discrimination was good in population with children affected by psychiatric disorders. For each item of the CDI, the child must choose between 3 statements the one that best describes his or her ideas and feelings in the past 2 weeks. Each item is rated from 0 (normal or absent) to 2 (severe) and half of the items are inverted to avoid perseveration effects. The sum of all the items ranges from 0 to 54 and the cut-off was set at 13 by Kovacs. The higher the score, the more pathological the condition.

#### The Revised Children's Manifest Anxiety Scale

This is a 37-item self-administered questionnaire for the quantitative evaluation of anxiety traits (i.e., the individual's stable and general tendency to experience anxiety) in children from 8 years of age (24). Cronbach alpha was 0.84. Test–retest reliability Pearson coefficients after a 6-month interval was 0.70. Convergent validity was found to be good. The RCMAS provides a total anxiety score (TA) as well as scaled scores on three anxiety subscales. The TA is based on 28 items which are divided into 3 anxiety subscales: (i) “physiological anxiety” (PA) consists of 10 items about somatic manifestations of anxiety such as sleep latency problems, nausea, and fatigue; (ii) “worry/oversensitivity” (IH) consists of 11 items measuring obsessive concerns about a variety of things, most of which are typically vague and ill-defined, as well as fears about being hurt or emotionally isolated), and (iii) “social concerns/concentration” (PC), being 7 items that measure distracting thoughts and fears that have a social or interpersonal nature. The remaining 9 items of the RCMAS constitute the lie subscale to assess the validity of responses. Because scores are derived from affirmative responses, a high score indicates a high level of anxiety or lie on that subscale. High scores on the subscales can represent different aspects of anxiety, which can be used to develop hypotheses about the origin and nature of the child's anxiety. The scale has been validated on children aged 6–19 and the psychometric properties have been reported (25). The thresholds for screening for anxiety traits correspond to the standardized mean plus one standard deviation (85th percentile), i.e., 60.0 (50.0 + 10.0) for the BP score and 13 (10.0 + 3.0) for each of the sub-scores (26).

#### The Conners Scales (for Parents and Teachers)

This scale is a reliable tool to assist the professional in the diagnosis of attention deficit hyperactivity disorder (ADHD) (27). This scale provides a complete rating of attention deficit with or without hyperactivity. This scale also assesses disorders frequently associated with ADHD, such as oppositional defiant disorder and conduct disorder. In this study, we used the long version of the Conners Parent Rating Scale (CPRS-R) and the short version of the Conners Teacher Rating Scale (CTRS-R) (28). The CPRS-R is an 80-item self-administered questionnaire, each item being rated from 0 (never) to 3 (often). The sum of the items ranges from 0 to 240. The CTRS is a 28-item self-administered questionnaire, each being also rated from 0 (never) to 3 (often). The sum of the items ranges from 0 to 84. For both CTRS and CPRS, the higher the score, the more severe the difficulties. This questionnaire was given to parents during the visit along with a cover letter for the teacher and a postage-paid return envelope.

#### The Pediatric Quality of Life Inventory Version 4.0 (PedsQL™ 4.0)

This self-assessment scale is an internationally recognized instrument for measuring health-related quality of life in the European Union (29). This is a 5-point Likert scale from 0 (Never) to 4 (Almost always). Items are reversed scored and linearly transformed to a 0-100 scale as follows: 0 = 100, 1 = 75, 2 = 50, 3 = 25, 4 = 0. Higher scores indicate better Quality of Life (QOL).

#### Statistical Analysis

All statistical analyses were performed using the Statistical Package for Social Sciences (SPSS) for Windows version V25.0 (IBM Corp., Armonk, NY). Descriptive statistics were produced in the forms of graphs, means, standard deviations, percentages, and 95% confidence intervals. The analysis of patients was conducted for the entire clinical group. Child self-reported scores of the PedsQL were compared, using one sample *t*-tests, to the norm scores of a healthy population sample and a general chronic health condition sample, defined by Varni and Burwinkle (30) as “a physical or mental health condition that has lasted or is expected to last at least 6 months and interferes with the child's activities.” Then we analyzed patients one by one, examining each patient with pathological scores. A patient was considered to have a deficit when they presented a score over 13 on the CDI scale was considered as a pathological score. Considering anxiety, a score over 60 on the RCMAS and a score over 13 on the subscale was considered pathological.

## Results

### Characteristics of the Cohort

Twelve children, 10 boys and 2 girls, were included in this study (Table 1). Mean age at inclusion was 12.8 years ± 2.3 [9; 15] while mean age at diagnosis was 8.8 years ± 2.6 [4.6; 14.3]. The main characteristics of each children is described in Table 1.

**Table 1:** Molecular and psychiatric characteristics of the 12 children from our cohort.

**Individuals**	**1**	**2**	**3**	**4**	**5**	**6**	**7**	**8**	**9**	**10**	**11**	**12**
Sex	M	F	M	M	M	M	F	M	M	M	M	M
Age	10	13	15	13	11	15	10	9	15	15	14	15
Molecular diagnosis	m.8306T>C (MT-TK)	Compound heterozygozity in *NDUFB3* c.410T>C p.(Try22Arg) and c.554G>T p.(Gly70*)	m.14849 (MT-CYB)	Complex III abnormalities without pathogenic variant identified	m.13513G>A (MT-ND5)	m.3243A>G (MT-TL1)	m13513G>A	m.15992A>T (MT-TP)	m.15992A>T (MT-TP)	m.3243A>G (MT-TL1)	m.3243A>G (MT-TL1)	m.11778G>A (MT-ND4)
Mode of Inheritance	Maternal inheritance	Autosomal recessive	Maternal inheritance	Unknown	Maternal inheritance	Maternal inheritance	Maternal inheritance	Maternal inheritance	Maternal inheritance	Maternal inheritance	Maternal inheritance	Maternal inheritance
Plasma lactic acidosis	No	Mild	No	Mild	No	Moderate	Mild	Moderate	Mild	No	No	No
**Psychiatric features**												
Anxiety			**+**					**+**		**+**		
Depression			**+**					**+**	**+**		**+**	
Psychiatric or psychological follow-up	**+**	**+**	**+**	**+**	**+**	**+**					**+**	**+**

*M, Male; F, Female; +, presence of a symptom; Lactic acidosis was classified according to increase from normal reference values (0.5–2.2 mmol / L), i.e., mild, moderate, and severe: ≥2, ≥3, ≥4-fold the normal, respectively; Anxiety was defined by a pathological score on the Revised Children's Manifest Anxiety Scale (RCMAS). Depression was defined by a pathological score on the Child Depression Inventory (CDI)*.

### Psychiatric Features

Main results regarding psychiatric features are given in Table 2. The psychiatric interviews evidenced that six children (50%) (individuals 4, 5, 6, 7, 8, and 9) were suffering from specific phobia while three children (25%) (individuals 3, 5, and 9) had obsessive and compulsive symptoms. Two children (child 6 and 11) (16.7%) reported having suffered from hallucinations. Two children (child 3 and 11) (16.7%) had behavioral disorders mainly consisting of intrafamilial aggressive behavior, and two children had sleep disorders (child 4 and 5). No child exhibited symptoms evocative of autism or delusional disorder. Eight children (72.7%) required a psychiatric or a psychological follow-up.

**Table 2:** Somatic and psychiatric characteristics of the studied population of children with mitochondrial disorder (*n* = 12).

**Characteristics**	
Age (years), mean ± sd [min; max]	12, 8y ± 2.3y [9; 15]
Male	*n* = 10 (83.3%)
Age at diagnosis	8.8 ± 2.6 y [4.6; 14.3]
**Level of education**	
Elementary school	*n* = 4 (33.3%)
Secondary school	*n* = 6 (50%)
High school	*n* = 1 (8.3%)
Grade repetition	*n* = 8 (66.7)
Special education	*n* = 4 (66.7%)
No schooling	*n* = 1 (8.3)
**Neurological features**	
– Ophthalmoplegia	*n* = 12 (100%)
– Distal myopathy	*n* = 12 (100%)
– Proximal myopathy	*n* = 1 (8%)
– Facial myopathy	*n* = 1 (8.3)
– Exercise intolerance	*n* = 9 (75%)
– Migraines	*n* = 4 (33.3%)
– Psychomotor regression	*n* = 4 (33.3%)
– Ataxia	*n* = 3 (25%)
Dystonia	*n* = 3 (25%)
Tremors	*n* = 2 (16.7%)
Spasticity	*n* = 1 (8.3%)
– Deafness	*n* = 2 (16.7%)
– Pseudo-strokes	*n* = 2 (16.7%)
– Phamarcoresistant epilepsy	*n* = 2 (16.7%)
– Continuous partial epilepsy	*n* = 1 (8.3%)
– Cardiomyopathies	*n* = 1 (8.3%)
– Optical atrophy	*n* = 1 (83%)
– Ptosis	*n* = 1 (83%)
– Swallowing	*n* = 1 (8.3%)
– Hypotonia	*n* = 1 (8.3%)
– Bulbar injury	*n* = 1 (8.3%)
**Lactic acidosis (LA) severity**	
– No LA	*n* = 6 (50%)
– Mild LA	*n* = 4 (33.3 %)
– Moderate LA	*n* = 2 (16.7%)
– Severe LA	*n* = 0
**Developmental milestones**	
Age at sitting	0.7y ± 0.1 [0.5; 0.8]
Independent walking	*n* = 10 (83.3%)
Age at walking	1.5 ± 0.4 y [1.1; 2.3]
Age at first words	1 y ± 0 [1; 1]
**Psychiatric scales**	
**EGF score**	
11–20	*n* = 1 (8.3%)
41–50	*n* = 2 (16.7%)
51–−60	*n* = 2 (16.7%)
61–70	*n* = 5 (41.7%)
71–80	*n* = 1 (8.3%)
81–90	*n* = 1 (8.3%)
	mean ± sd [min; max]
BPRS score	44.6 ± 11.4 [29; 63]
CDI score	11.8 ± 7.0 [3; 24]
RCMAS total score	54.7 ± 10.7 [33; 78]
– Physiological anxiety (PA)	11.6 ± 3.6 [5; 17]
– Worry/hypersensitivity (IH)	10.5 ± 3.2 [6; 18]
– Social concerns/concentration (PC)	11.7 ± 2.4 [8; 17]
– Lie	11.8 ± 3.5 [6; 18]
**Conners score**	
**Parental score**	
CPRS-R score total	9.7 ± 6.5 [0; 21]
Index ADHD	17.0 ± 9.1 [0; 29]
Index agitation/Impulsivity	7.5 ± 4.9 [0; 15]
Index emotional lability	2.2 ± 2.1 [0; 6]
**Teacher's score**	
CTRS-R total score, mean ± sd [min; max]	12.5 ± 10.1 [1; 33]
Opposition, mean ± sd [min; max]	0.6 ± 1.2 [0; 4]
Inattention, mean ± sd [min; max]	5.8 ± 4.3 [0; 11]
Hyperactivity, mean ± sd [min; max]	0.6 ± 1.0 [0; 3]
ADHD	5.5 ± 5.4 [0; 16]
– PedsQL score, mean ± sd [min;max]	58.5 ± 15.5 [39; 96]
– Physical health summary score, mean ± sd [min;max]	50.3 ± 22.6 [15.6; 100]
– Psychosocial health summary score, mean ± sd [min;max]	62.9 ± 15.1 [36.7; 93.3]

*sd, standard deviation; %, percentage; min, minimum; max, maximum; y, years; n, number; BPRS, Brief Psychiatric Rating Scale; CDI, Child Depression Inventory; CPRS-R, Conners Parent Rating Scale; CTRS-R, Conners Teacher Rating Scale; CGAS Global Assessment of Functioning; PedsQL, Pediatric Quality of Life Inventory; RCMAS, Revised Children's Manifest Anxiety Scale*.

Using the CDI, four children (33.3%) were diagnosed with depressive symptoms. Nevertheless, the mean CDI score of the cohort was 11.8, which is below the depression threshold set at 13.

Six children (50%) reported anxiety symptoms during the psychiatric interview, and three children (25%) were suffering from anxiety according to the RCMAS scale. The lie score was, however, the higher subscore of this scale in the cohort, with a mean score of 11.8 ± 3.5. Three children had a lie score >13, while having a non-pathological score on the whole scale.

Five children (41.7%) had mild symptoms or some difficulties in social, occupational, or school functioning according to the CGAS scale, with a score between 61 and 70. Regarding the scores obtained on the BPRS (Table 3), seven children (58.3%) had pathological scores for anxiety items, while 6 children (50.0%) had pathological scores for suspiciousness items as well as psychomotor retardation items. Finally, five children (41.7 %) had pathological scores for depressive mood items, but none of the children had a pathological score for somatic concerns.

**Table 3:** Psychiatric characterization of the cohort using the brief psychiatric rating scale.

**Brief psychiatric rating scale items**	**Mean scores mean ± sd** ** [min; max]**	**Individuals with pathological Scores, *n* (%)**
Somatic concerns	3.8 ± 2.1 [1; 7]	0 (0)
Anxiety	3.8 ± 2.1 [1; 7]	7 (58.3)
Emotional withdrawal	1.8 ± 1.3 [1; 4]	2 (16.7)
Conceptual disorganization	2.1 ± 1.5 [1; 5]	3 (25)
Guilty feelings	2.3 ± 1.5 [1; 6]	2 (16.7)
Tension	2.4 ± 1.9 [1; 6]	4 (33.3)
Mannerisms and posturing	1.9 ± 1.8 [1; 7]	1 (8.3)
Grandiosity	1.7 ± 1.4 [1; 5]	2 (16.7)
Depressive mood	3.8 ± 2.0 [1; 7]	5 (41.7)
Hostility	2.0 ± 2.3 [1; 7]	2 (16.7)
Suspiciousness	3.0 ± 2.0 [1; 6]	6 (50.0)
Hallucinatory behavior	1.9 ± 1.9 [1; 7]	2 (16.7)
Motor retardation	4.0 ± 2.4 [1; 7]	6 (50.0)
Uncooperativeness	1.8 ± 1.5 [1; 5]	2 (16.7)
Unusual thought contents	1.4 ± 1.2 [1; 5]	1 (8.3)
Blunted affect	2.2 ± 1.6 [1; 5]	3 (25)
Excitement	2.5 ± 2.2 [1; 7]	3 (25)
Disorientation	2.3 ± 2.0 [1; 7]	3 (25)

*%, percentage; sd, standard deviation; min, minimum; max, maximum*.

The Conners scores, quantifying ADHD, were low in our cohort, with a mean score of 9.7 ± 6.5 [0; 21] and 12.5 ± 10.1 [1; 33] for parents and teachers respectively.

The mean PedsQL score, evaluating the quality of life, was low: 58.5 ± 15.5 [39.0; 96.0]. The quality of life score was higher when reporting psychosocial health compared to physical health (62.9 vs. 50.3%). Child proxy reports revealed a lower overall QOL score and lower physical, psychosocial, and school subscales score, but did not show any significant difference in terms of emotional subscales compared to the general population. Compared to children with chronic illnesses, children with MD reported a lower overall quality of life score and lower scores in physical and social subscales. The results regarding the quality of life are shown in detail in Table 4.

**Table 4:** Data on children's quality of life assessed using the Pediatric Quality of Life Inventory (PedsQL,) version 4.0, compared to standards obtained from a normal population and from a population of chronically ill children.

**PedsQL scales**	***n***	**Mean**	**sd**	**Mean in a population of healthy individuals^**#**^**	**Student's *t* (*df* = 11)**	***p***	**Mean in a population of chronically ill individuals^**#**^**	**Student's *t* (*df* = 11)**	***p***
Total	12	58.5	15.5	81.1	−5.04	≤0.001^*^	71.6	−2.92	0.01^*^
Physical	12	50.3	22.6	85.6	−5.41	≤0.001^*^	78.0	−4.30	0.001^*^
Psychosocial	12	62.9	15.1	78.7	−3.61	0.004^*^	68.0	−1.16	0.27
Emotional	12	66.3	19.8	74.8	−1.50	0.16	64.9	0.25	0.81
Social	12	61.7	15.1	83.5	−4.99	≤0.001^*^	72.3	−2.42	0.03^*^
School	12	60.9	20.1	77.8	−2.92	0.01^*^	66.8	−1.03	0.33

# *Norms Varni et al. (29) (the PedsQL as a population health measure);*

* *the correlation is significant to the level 0.05 (bilateral); n, number; sd, standard deviation; df, degree of freedom*.

The results of each of the twelve children for the different tests and scales are given in Table 5.

**Table 5:** Characteristics and results of the psychiatric rating scales for each child and adolescent included in the study (*n* = 12).

**Individuals**	**1**	**2**	**3**	**4**	**5**	**6**	**7**	**8**	**9**	**10**	**11**	**12**
Sex	M	F	M	M	M	M	F	M	M	M	M	M
Age	10	13	15	13	11	15	10	9	14	15	14	15
BPRS	62	44	63	29	41	45	47	58	33	37	44	32
GAF	61–70	61–70	51–60	71–80	11–20	41–50	41–50	61–70	61–70	61–70	51–60	81–90
PedsQL	75	62	39	63	52	41	64	46	52	54	58	96
CDI	10	10	22^*^	6	5	7	8	14^*^	21^*^	12	24^*^	3
RCMAS	49	48	78^*^	54	49	54	52	62^*^	60	61^*^	56	33
CPRS	14	13	21	4	2	6	5	15	9	10	17	0
CTRS	11	14	33	10	16	NA	13	3	1	8	27	1

*M, Male; F, Female; BPRS, Brief Psychiatric Rating Scale (Scores ranging from 18 to 126; scores >72 define pathological deviation); CGAS, Global Assessment of Functioning (scale gradated along a continuum in increments of 10, the gradation goes from 1 to 10 for the sickest individuals to 90–100 for the healthiest individuals); CDI, Children Depression Inventory (scores range from 0 to 54 with a cut-off of 13); RCMAS, Revised Children's Manifest Anxiety Total T Score (cut-off > 60); CTRS-R, Conners Teacher Rating Scale; CPRS-R, Conners Parent Rating Scale (sum of the items ranges from 0 to 84; the higher the score, the more severe the difficulties); PedsQL, Pediatric Quality of Life Inventory Version 4.0 (scores range from 0 to 100; higher scores indicate better Quality of Life)*.

* *and bold values represents pathological score*.

## Discussion

Our results confirm data found in the literature on psychiatric features in children affected with MD (12), in particular anxiety and depression.

### Anxiety Symptoms

A quarter of the children in our cohort (*n* = 3, 25%) presented anxiety symptoms as measured by the RCMAS and the mean score of the cohort using this scale was 54.7, which is close to the cut-off of 60 for anxiety. This result is close to that of Eom and Lee (13) who found that 29% of children and adolescents with MD were anxious and depressed whereas in the general pediatric population anxiety disorders have a prevalence of 6 to 18% (31). However, our study is the first, to our knowledge, to assess anxiety in children with MD using a specific anxiety scale.

We also tested for anxiety using a General Psychiatric Questionnaire (BPRS) and found that the majority of children in our cohort (58.5%) had pathological scores for anxiety items.

In addition, on the basis of semi-structured interviews with a child psychiatrist, anxiety symptoms were evidenced in half of the children (*n* = 6, 50%). There are therefore significant differences between the assessment of anxiety by scales, in particular by the RCMAS and assessment through an interview.

This discrepancy can be explained by high scores obtained on the RCMAS “lie” scale which aims to identify acquiescence, social desirability, and children's falsification of some responses. Indeed, this lie scale reveals more of children's defense mechanisms against anxiety than the anxiety itself (32). This confirms our clinical hypothesis that children with DM would have a strong desire to minimize their symptoms in order to reassure their family and maybe even medical staff. In connection with this, it should be noted that children in this study were very frequently accompanied by their mother during interviews and that the RCMAS items were almost always completed in the presence of mothers. This hypothesis has to be verifiy.

In our cohort, mitochondrial disease had been maternally transmitted in 10 cases out 12 (i.e., caused by a mtDNA variant), was recessively inherited in one case while the molecular basis was not determined in the remaining case (Table 1). Each mother, however, mentioned a marked guilt and it was difficult for them to talk about their child's pathology because of their emotional load.

Interestingly, studies that analyzed depression in mothers of children with MD found that 42 à 65% of them had significant levels of depression (13, 33). To note, high rates of depression in mothers are known to impact youth mental health negatively (34).

In addition, DM induce situations that are particularly likely to provoke anxiety for families and children, due to the unpredictable course of these diseases and but also because of their gravity and their impact on daily life. Also, some of the children in our study had siblings and/or mothers who were also suffering from a DM. The children were frequently aware of this and were likely to minimize their symptoms in order to reassure their mothers.

Finally, the prevalence of specific phobias was very high in our cohort (*n* = 6, 50%), much higher than that of the general population, where such phobias are only found in around 8% (35) of children. This difference can be explained by the fact that data were collected during semi-structured interviews without any specific scale. This high prevalence rate of phobias is nevertheless notably and needs to be further studied using specific tools.

### Depressive Symptoms

Using the CDI scale, a diagnosis of depression was made in one third of the children in our cohort (*n* = 4, 33.3%) and the mean score (11.8) of the cohort was close to the cut-off (13). Using the BPRS general psychiatric scale, 41.7% (*n* = 5) of the children had pathological scores for the item “depression.” In comparison, depressive disorders affect around 15–20% of the general population regardless of age, and nearly 2 and 8% of prepubescent children and adolescents are suffering from depression, respectively (36).

In a recent review of the literature (12), we found that depressive symptoms were present with a prevalence ranging from 14.3 to 38.9% in children and adolescents with MD (37, 38). In the different studies reviewed, the diagnosis of depression was either clinical or based on the use of the Child Behavior Check List (CBCL) (38) or the Hamilton Depression Scale (HDRS) when the children were over 14 years old (37). In our study, the use of a specific depression scale (i.e., CDI), therefore reinforces the fact that children and adolescents with mitochondrial pathologies have an increased risk of suffering from depression.

Beyond the specificity of MD, children and adolescents with chronic pathologies are more at risk of presenting psychiatric symptoms, and in particular anxiety and depressive symptoms (39, 40). Indeed, we observed that the prevalence of depression in children and adolescents with MD was substantially similar to that found in other chronic health conditions. For instance, Denny et al. (41) showed that 40% of individuals in a population of 125 adolescents affected with chronic diseases resulting in an impact on their socialization, had depressive symptoms (41). Another study, involving 670 individuals with cystic fibrosis aged 12 to 64 years, showed a 9.6% and 20.6% prevalence of depression and anxiety, respectively (39).

As regards more specifically mitochondrial pathologies, it has been suggested that anomalies of energy metabolism in the central nervous system could explain the mood disorders observed in children and adolescents (37). However, the etiology of depression and anxiety in young people with MD is complex and likely involves the biological and genetic bases of these diseases (14). Conversely, mitochondrial dysfunctions have been found in people with depression or other psychiatric disorders, such as bipolar disorder or schizophrenia (42, 43).

In sum, these data, including those reported in this study, appear to report a high sensitivity to psychiatric disorders, in particular depressive syndromes, in children and adolescents with MD. This risk factor probably combines specific brain dysfunction due to mitochondrial abnormalities with the fact of being affected with a chronic disease.

### Functioning and Quality of Life of Children With MD

A significant proportion of children in our cohort (*n* = 5, 41.7%) had a score between 61 and 70 on the CGAS scale which assesses the psychological functioning, social adaptation, and activity of an individual.

These results show a real deterioration in the overall functioning of children and adolescents with MD and are consistent with those provided by Eom and Lee (13) who showed that 80% of their cohort of 70 children with MD had major difficulties in their daily life (13).

Along the same lines, the mean quality of life score (i.e., PedsQL score) was low in our cohort mainly revealing a greater deterioration in physical health than in psychosocial health (62.9 vs. 50.3%). In addition, children with MD showed a lower overall quality of life score and lower scores for the physical and social activities subscales compared to the norms established in children with chronic illnesses as a whole (29).

Our results therefore confirm a major deterioration in the quality of life of children and adolescents with MD corroborating what has previously been shown by several studies (44, 45).

### Attention Deficit and Hyperactivity

Conners test scores were low in our cohort, indicating that the children with MD we tested did not have ADHD. This is in contradiction to what Eom and Lee (13) showed in a cohort of 70 Korean children with MD. This discrepancy could be explained by a difference in methodology in the assessment of ADHD in the two studies. Indeed, Eom and Lee used a Korean version of the Child Behavior Check List questionnaire without specifically assessing ADHD using a specific questionnaire like the Conners one.

### Other Psychiatric Symptoms

In this exploratory study, we used the BPRS General Psychiatric Scale to more easily assess psychiatric symptoms that are little known and little studied in MD. According to the results of the BPRS, half of the children in our cohort (*n* = 6, 50%) had pathological scores on the items of suspicion and on those of psychomotor delay while none had pathological scores for somatic problems. These results were unexpected, especially with regard to the somatic problems which were found to be preponderant in a recent study carried out on a cohort of adults with MD (44). Again, these discrepancies may support our hypothesis that children tend not to talk about their illness and downplay their symptoms to alleviate their own anxiety and that of their family. A majority of children (*n* = 8, 72.7%) required a follow-up either by a psychiatrist or by a psychologist. This follow-up was in most cases initiated by the family and had been often put in place without the agreement or approval of the child's pediatrician. This is, however, consistent with the large proportion of children in our cohort who presented with psychiatric symptoms, particularly anxiety and depression. This reflects above all the intensity of the psychiatric symptoms presented by children with DM in their daily life and the need to set up systematic psychotherapeutic monitoring in this context.

However, we noted that three children presented with anxiety and depressive symptoms but did not have any psychological or psychiatric follow-up. All of this reinforces the need for early identification of psychiatric disorders in children with MD, in particular those, as in our cohort, for whom the somatic consequences of the disease are not major. Finally, we thought that lactic acidosis could have an influence on the presence of psychiatric disorders, but such a relationship was not demonstrated in our sample.

### Limitations of the Study

The first limitation of this study concerns the level of severity of MD. The children included had the ability to express themselves and communicate and therefore presented a moderately disabling form of MD, similarly to those included in the studies performed by Koene et al. (37) and Morava et al. (38). Nevertheless, many of them had psychiatric symptoms and were impacted by the disease in their daily lives.

The second limitation of the study is the sample size, which was limited to 12 individuals. However, this number adequately represents the recruitment of individuals affected with a rare disease in a single reference center over one year, after having excluded very severe forms of the disease. By comparison, the 4 previously published studies on psychiatric disorders in children with DM included 18 to 112 individuals (13, 37, 38, 46). However, the study involving 112 children (46), did not specifically target MD but concerned children affected with progressive intellectual and neurological deterioration. To compensate for the small number of individuals of our sample, we have tried to be exhaustive in our analyses of psychiatric disorders by using numerous scales and by performing a systematic psychiatric assessment.

Also, all of the psychiatric symptoms that the authors found in these youth are known to be present at higher rates in youth with chronic illnesses in general (39, 41, 47, 48).

## Conclusion

Our study highlights the need to pay particular attention to the psychiatric symptoms of children and adolescents presenting with MD, especially depression and anxiety. Regular psychiatric assessments are essential because the disease occurs in individuals with developing brains. In addition, MD are chronic and progressive pathologies, which require regular reassessment. In terms of monitoring psychiatric disorders, we suggest an annual reassessment by a child psychiatrist. The chronicity of the disease should lead to a particular attitude toward not only the child, but also his or her family. Psychological support should be offered not only to sick children but also to their families.

Beyond the psychiatric assessment, a more comprehensive assessment of the child's neuropsychological functioning could be of real interest in MD, with by example intelligence tests. Indeed, the early vulnerability of children's executive functions is now proven in many clinical contexts in which developing pre-frontal circuits are exposed to a developmental anomaly or to acquired brain damage (49).

Paradoxically, disorders of executive functions, which appear to be linked to anxiety and depression in children and adults (50, 51), have been very little studied in individuals affected with MD (52).

## Data Availability Statement

The raw data supporting the conclusions of this article will be made available by the authors, without undue reservation.

## Ethics Statement

The studies involving human participants were reviewed and approved by French Committee for the Protection of Subjects involved in Biomedical Research. Written informed consent to participate in this study was provided by the participants' legal guardian/next of kin.

## Author Contributions

ER, MB, PD, and AR design of the study. ER, TL, NP, PA, VP, CP, MB, PD, and AR data analysis. ER, DB, PD, and AR article writing. ER, TL, NP, MB, CP, EC, PA, VP, PV, PD, DB, and AR revision of the manuscript. All authors contributed to the article and approved the submitted version.

## Conflict of Interest

The authors declare that the research was conducted in the absence of any commercial or financial relationships that could be construed as a potential conflict of interest.
